# Non-disruptive in vitro monitoring of cellular states with cell-free DNA methylation

**DOI:** 10.1186/s13059-026-03996-1

**Published:** 2026-02-17

**Authors:** Anja Hess, Alexander Kovacsovics, Fabian Bachinger, Ludovic Vallier, Helene Kretzmer, Alexander Meissner

**Affiliations:** 1 Max Planck Institute for Molecular Genetics, Berlin, Germany; 2 Department of Biology, Chemistry and Pharmacy, Freie Universität Berlin, Berlin, Germany; 3 Berlin Institute of Health, BIH Center for Regenerative Therapies, Charité- Universitätsmedizin, Berlin, Germany; 4 Digital Health Cluster, Digital Engineering Faculty, Hasso Plattner Institute for Digital Engineering, University of Potsdam, Potsdam, Germany

**Keywords:** Cell-free DNA, Cell identity, Liquid biopsy, Monitoring, Bioreactor, Hepatocytes, Stem cells

## Abstract

Individual CpG methylation signatures characterize each mammalian cell type, thereby offering a powerful readout for clinical and diagnostic applications. Here, we apply this principle to in vitro cellular systems, using cell-free DNA released into the culture medium to track epigenomic responses to small-molecule treatments and bioreactor-based directed differentiation. Coupled with Oxford Nanopore sequencing, our approach enables rapid assessment of global CpG methylation dynamics and enhancer states. This strategy provides a streamlined method that can be readily applied and customized for non-disruptive monitoring of various in vitro screening modalities.

**Supplementary Information:**

The online version contains supplementary material available at 10.1186/s13059-026-03996-1.

## Background

All mammalian cell types exhibit distinct CpG methylation patterns, as shown by extensive genome-wide profiling, including single-base resolution maps of purified cell types [1–7]. Cell-free DNA (cfDNA), primarily derived from mononucleosomal fragments, retains these methylation marks, preserving information about the cell of origin [8–10]. This property has enabled cfDNA-based analyses for cancer detection and monitoring, as well as for many other diseases where cell-type specific knowledge is relevant [11–16]. Similar principles have also been applied to detect somatic contamination in human preimplantation embryo cultures [17, 18].

## Results and discussion

**Fig. 1:**
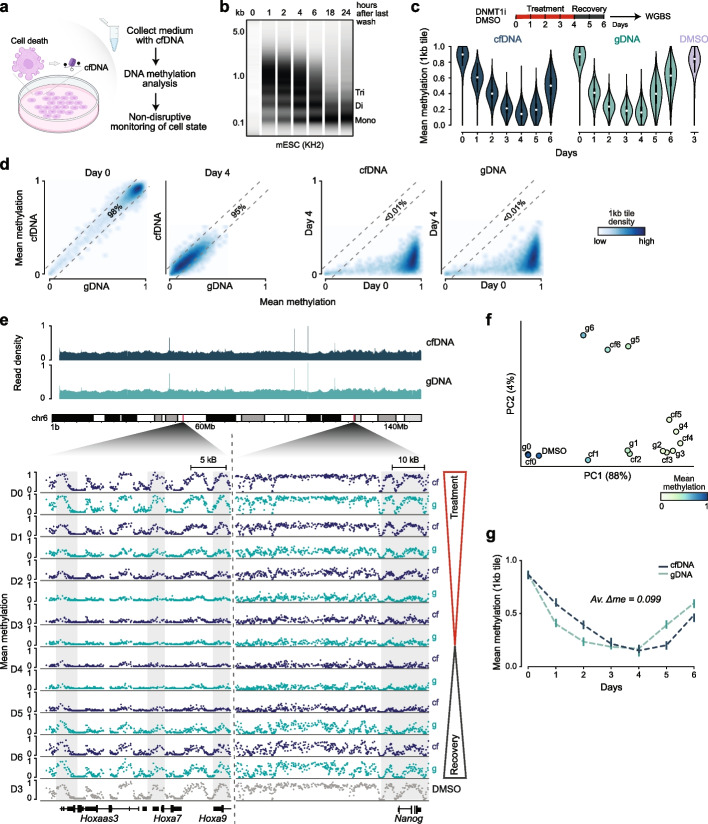
Cell-free CpG methylation enables non-invasive monitoring of global epigenetic dynamics in vitro. **a** Schematic overview of a cell culture system where dying cells release cell-free material, including nucleosomal DNA fragments, into the medium. Medium collection and recovery of cell-free (cf) DNA, followed by methylation analysis, enables non-invasive monitoring of cell states via CpG methylation dynamics. Graphic created with BioRender. **b** Electrophoresis traces of cfDNA isolated from mouse embryonic stem cell (ESC, KH2, passage 19) culture supernatants collected at 1–24 h after the last wash. One representative replicate is shown (*n* = 3). **c** Experimental design: Murine ESCs were treated with the DNMT1 inhibitor GSK-3484862 (GSK) for four days, followed by a two-day recovery phase. Violin plots show mean CpG methylation across 1 kb tiles for cfDNA and matched gDNA libraries, compared to DMSO control (day 3). *n* = 2,024,681 1 kb tiles covered in all samples. **d** Correlation plots comparing 1 kb-tile mean CpG levels between cfDNA and gDNA at day 0 and 4 (left), and between day 0 and 4 in cfDNA or gDNA (right). Darker color indicates higher CpG density; striped lines indicate 0.1 methylation difference between the conditions. Percentage of tiles with methylation difference ≤ 0.1: cfDNA vs. gDNA: 98% (day 0), 95% (day 4); day 0 vs. 4: < 1% (cfDNA), < 1% (gDNA). Pearson R-values: cfDNA vs. gDNA: 0.95 (day 0), 0.85 (day 4); day 0 vs. 4: 0.41 (gDNA), 0.40 (cfDNA), *p* < 0.0001 for all correlations. **e** Read density plots for chromosome 6 (top) and representative browser tracks showing local methylation levels on CpG level across the inhibitor treatment and recovery phase for cf- and gDNA (bottom). **f** Principal component analysis (PCA) of mean CpG methylation (1 kb tiles) for each time point. Dot color indicates CpG level. **g** Line plot showing mean CpG methylation dynamics in cfDNA and gDNA across the time course. The average delta methylation between the two conditions is 0.099

To explore the broader utility of cfDNA methylation, we investigated whether basal levels of cell death produce sufficient cfDNA to non-invasively capture the methylation landscape of the respective cell culture model. We further explored whether this signal reflects the dynamic cell state changes following perturbation or during directed differentiations (Fig. 1a).

To quantify the temporal dynamics of release and subsequent recovery of cfDNA, we cultured different mouse embryonic stem cell lines and seeded them to reach approximately 500,000 cells per well within 24 h (Additional file 1: Table S1). After thorough washing, we added 300 µl of fresh medium and collected ~ 280 µl at defined time points over the next 24 h. The actual cells from each well were used for fluorescence activated cell sorting (FACS) to assess apoptosis rates using AnnexinV staining (Additional file 2: Fig. S1). From the collected medium, we removed debris through a brief centrifugation, isolated cfDNA and then assessed both its concentration and fragment size distribution. We confirmed that the cfDNA was nucleosomally sized and absent from our negative gDNA and medium controls (Fig. 1b; Additional file 3: Extended Data Fig. 1a, b). All time points yielded nucleosomal cfDNA, with 3–41 ng at 1 h and 61–192 ng at 24 h (Additional file 1: Table S2), which are in a suitable range for the preparation of whole genome bisulfite sequencing (WGBS) libraries (Additional file 3: Extended Data Fig. 1c, d). We also observed a progressive shift in cfDNA fragment size from approximately 1 kb at 1 h to ~ 160 bp after 24 h, which likely reflects continuous endogenous nuclease digestion in the media leading to mononucleosomal fragment sizes (Fig. 1b; Additional file 3: Extended Data Fig. 1d, e).

Next, we investigated the information content and potential biases of cfDNA from in vitro cultures by treating KH2 cells with the DNMT1 inhibitor GSK-3484862 [19] (2.5 µM) for four days, followed by a two-day recovery period. In parallel, we collected matched genomic DNA (gDNA) from the same populations. To standardize cfDNA release time and enrich for recently shed material, we washed the cells one hour prior to media collection (Fig. 1c). From 1.6–3.4 million cells, we recovered an average of 37–62 ng cfDNA (Additional file 1: Table S3) and successfully prepared WGBS libraries, achieving comparable genome-wide CpG coverage for all samples of cf- and gDNA (Additional file 3: Extended Data Fig. 1f-h). cfDNA methylation faithfully recapitulated the expected global dynamics of DNMT1 inhibitor-induced loss and subsequent recovery of methylation over the time course (Fig. 1c). Correlation analyses at the 1 kb-tile level confirmed the accuracy of our cfDNA-derived methylation profiles, and representative loci highlight that comparable information, including inhibitor-induced changes, can be captured without disrupting the cultured cells (Fig. 1d-f). Of note, we observe a ~ 24 h offset between global methylation dynamics of cfDNA and gDNA: cfDNA methylation values remained marginally higher during inhibitor treatment and rose more slowly after withdrawal compared to gDNA (Fig. 1g; Additional file 3: Extended Data Fig. 1i). We interpret this delay as a consequence of apoptosis dynamics, which likely enrich cfDNA from historical cells that were not proliferating anymore prior to release of genomic material into the media [20]. Taken together, these findings demonstrate that cfDNA methylation profiling in vitro enables non-disruptive tracking of CpG methylation dynamics.

**Fig. 2:**
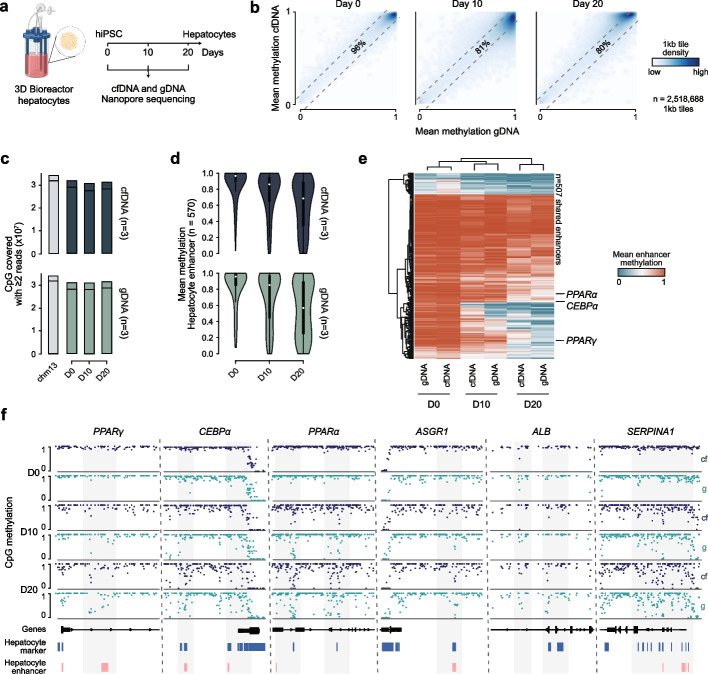
cfDNA methylation profiling captures 3D hepatocyte differentiation dynamics in bioreactors. **a** Schematic overview of 3D bioreactor-based forward programming of human iPSCs into hepatocytes and proposed cfDNA monitoring via Nanopore sequencing. Graphic created with BioRender. **b** Correlation plots comparing 1 kb-tile mean CpG methylation levels in cfDNA and gDNA at day 0, 10, and 20. Darker colors indicate higher CpG density; striped lines indicate a 0.1 methylation difference between the conditions. Percentage of tiles with methylation difference ≤ 0.1: cfDNA vs. gDNA: 96% (day 0), 81% (day 10), 80% (day 20). Pearson R-values: cfDNA vs. gDNA: 0.93 (day 0), 0.83 (day 10), 0.89 (day 20); *p* < 0.0001 for all correlations. *n* = 2,518,688 1 kb tiles (covered by all samples). *N* = 3 (biological replicates merged for display). **c** Bar plots show the number of CpGs in cf- and gDNA libraries for day 0, 10, and 20; number of CpGs in chm13v2 is plotted in grey on the left for reference. Line indicates count for autosomal CpGs (chm13v2) covered by ≥ 2 reads (2X). **d** Violin plots show the mean CpG methylation in 570 hepatocyte enhancers across the differentiation time course (enhancers from [4], further required to lose methylation from pluripotent state to hepatocytes, i.e. iPSC methylation > 0.1 and < 0.1 in primary human hepatocytes) across the time course in cfDNA and gDNA). *N* = 570 enhancers. *N* = 3 (biological replicates merged for display). **e** Heatmap of mean CpG methylation across 507 (number of regions shared among all samples, out of 570) hepatocyte enhancers over time in cfDNA and gDNA. Samples are clustered hierarchically by similarity. Selected associated genes are highlighted. *N* = 3 (biological replicates merged for display). **f** Representative browser tracks showing mean CpG methylation in select hepatocyte enhancer (red) and marker (blue) regions across the time course in cfDNA and matched gDNA. Tracks of biological replicates (*n* = 3) were merged for display

After confirming the accurate global monitoring, we aimed to achieve two additional objectives: to demonstrate the utility of our assay in a more complex setting and to evaluate the feasibility of a faster turn-around. Here we decided to use Oxford Nanopore Technology (ONT) sequencing, which enables data acquisition within hours of sample collection [21]. As a proof-of-concept, we chose a 20-day differentiation protocol of human induced pluripotent stem cells (iPSCs) towards functional hepatocytes [22], cultured in an established 3D bioreactor system that yields Albumin-positive hepatocytes (Fig. 2a; Additional file 3: Extended Data Fig. 2a). We collected matched cf- and gDNA from the bioreactor cultures at days 0, 10, and 20, prepared Nanopore sequencing libraries using a standard native barcoding protocol, and ran them on PromethION flow cells. Each library was sequenced to approximately ~ 2X genomic coverage, with a total turnaround time of 380 min from sample collection to data generation (Additional file 3: Extended Data Fig. 2b). We basecalled the Nanopore data using Dorado [23] and aligned to the chm13v.2 human reference genome. We recovered native fragment sizes at read level, detecting the expected short, nucleosomally patterned cfDNA fragments that were distinct from longer gDNA fragments (Additional file 3: Extended Data Fig. 2c). As before, cfDNA and gDNA captured similar numbers of CpGs at comparable read coverage and showed a clear correlation in methylation values at all time points (Fig. 2b, c; Additional file 3: Extended Data Fig. 2d, e).

The ONT pipeline also captures 5-hydroxymethylation, which shows similar high correlations across our samples (Additional file 3: Extended Data Fig. 2f). To assess whether we could monitor the acquisition of hepatocyte identity using cfDNA, we analyzed the methylation state of select hepatocyte enhancers (defined using the data by Loyfer et al. [4]) as well as hepatocyte marker genes (see Methods) and observed progressive demethylation over the 20-day time course, in both cf- and gDNA (Fig. 2d; Additional file 3: Extended Data Fig. 3a, b). As in our earlier experiments, we noted a slight delay in the cfDNA signal; however this was not strong enough to affect an averaged unsupervised hierarchical clustering (Fig. 2e; Additional file 3: Extended Data Fig. 3c). We further examined key hepatocyte regulators and markers (*PPARG*, *CEBPA*, *PPARA, ASGR1*, *ALB* and *SERPINA1*) and observed gradual enhancer demethylation in both cf- and gDNA (Fig. 2f; Additional file 3: Extended Data Fig. 3 d). These findings demonstrate that Nanopore sequencing of cfDNA recovered from bioreactor media is sufficient to detect methylation signatures of evolving cell fates, including those associated with metabolic maturation.

## Conclusions

Non-invasive cfDNA methylation profiling from in vitro culture media enables high temporal-resolution tracking of dynamic epigenetic changes without disrupting the system. By capturing both global and focal methylation dynamics, this approach offers a powerful tool for monitoring cell fate transitions. Once a DNA methylation reference is available a cost-efficient and targeted panel should facilitate higher throughput screening modalities. As the approach relies solely on accessible media, it should be applicable across diverse in vitro models, potentially offering broad utility across various applications in both basic research and translational settings.

## Methods

### Cell culture

#### hiPSC lines

FSPS13B cells were derived and grown as previously described [24, 25] The lines were regularly tested negative for mycoplasma. *mESC lines.* Murine ESC (mESC) lines KH2, F1G4, and V6.5 were acquired as described [26–28] and initially MEF-depleted with TrypLE (Gibco, 12604021) at 37 °C for ten minutes followed by 60 min settling time and supernatant collection, frozen down and cultured feeder-free for a maximum of one additional passages for subsequent experiments. All mESCs were cultured on 0.2% gelatin in knockout DMEM medium (Gibco, 10829018) with 15% FBS, 1% penicillin/streptomycin (ThermoFisher Scientific, 15140122), 1% glutamine (ThermoFisher Scientific, 35050038), 1% non-essential amino acids (NEAA, Gibco), 0.055 mM β-mercaptoethanol (Gibco, 21985023) and leukemia inhibitory factor (LIF, generated in house). Splitting was performed with TrypLE (Gibco, 12604021). All lines were regularly tested negative for mycoplasma. *Other cultures for cfDNA characterization.* Primary CD1 mouse embryonic fibroblasts (MEFs, derived in house) were grown on 0.2% gelatine in mESC medium without LIF.

#### Human 3D forward programming bioreactor hepatocytes

FSPS13B hiPSCs were differentiated into forward programming hepatocytes as described [22], with slight modifications to the protocol: cells were cultured in 3D bioreactors (ReproCell ABLE Biott, ABBWVS03A-6), culture was fully xeno- and Matrigel-free, and Doxycyclin was removed at day 10.

#### cfDNA-monitoring culture conditions

All cultures were free of ambient cfDNA-sources, such as mouse tumor-derived matrices (Matrigel, Geltrex) or human cell line-conditioned medium for growth factor supply. Absence of (cf)DNA in medium was confirmed by gel electrophoresis prior to all experiments.

#### cfDNA collection, isolation and quantification

*Collection.* Medium was sampled from cell culture supernatant (SN) of 6-, 24-, or 96-well-plates or a bioreactor, tilting plastic ware 30 degrees, keeping the tip distant from cells or organoids. SN was centrifuged at 300 × *g* for 3 min to remove cellular debris, and approximately ¾ of the upper SN were transferred to a fresh tube. *Isolation.* SN was treated with Proteinase K (InnuScreen GmbH, 845-CH-0010006, stock concentration 22 mg/mL) for 1 h or overnight at 37 °C at 8.3 µl per 100 µl medium, and next purified with the DNA Clean & Concentrator Kit (Zymo, D4004) mixing SN with DNA binding buffer at ratio 1:5. gDNA was isolated with the PureLink Genomic DNA kit (Invitrogen, K182001) according to manufacturer’s instructions. *Quantification and size profiling.* DNA was quantified and profiled using the Qubit HS (for cfDNA) or BR (for gDNA) dsDNA/ssDNA assays (Invitrogen, 10616763) on a Qubit4 fluorometer (Invitrogen, 16223001), or through semiautomated gel electrophoresis using the High Sensitivity D5000-, and Genomic DNA ScreenTapes (all Agilent, 5067–5592/3 and 5067–5365/6) on a TapeStation 4150 device (Agilent, G2992AA), raw data and gel images were generated using the Agilent Software Package (2200 TapeStation Controller Software, and TapeStation Analysis Software).

#### DNMT1-Inhibitor treatment

mESCs were treated with 2.5 µM DNMT1-Inhibitor GSK-3484862 [19] (MedChem, HY-135146) for four days, followed by a two-day recovery period. Cells treated with equal volume of DMSO served as a negative control. Each day, cells were washed with DPBS(-/-) one hour before cfDNA collection, and cfDNA and matched gDNA were collected from the same well.

#### Time series for cfDNA collection with FACS apoptosis quantification

For time series, cells were washed twice with DPBS(-/-) to remove accumulated cfDNA and the well was replenished with fresh medium. A supernatant sample taken immediately afterwards served as time point 0. For the Staurosporine positive control, cells were washed twice with DPBS(-/-) to remove all remaining cfDNA and incubated in the presence of 1 µM Staurosporine (Sigma-Aldrich, 569396) overnight. SNs were then incubated for 1,2,4,16,18, or 24 h for cell-free DNA analysis and cells from the same well were used for FACS: briefly, medium was removed and cells were washed with DPBS(-/-) incubated with TrypLE (Gibco) for 7 min at 37 °C, dissociated in FACS buffer (10 mM HEPES, 140 mM NaCl, 2.5 mM CaCl_2_, pH 7.4), and stained with the AnnexinV conjugate (Invitrogen, A23204) according to the manufacturers instructions, and measured on a FACSAria II or Celesta (both Beckton Dickinson). The same protocol was used to quantify apoptosis in cells during the DNMT1i-treatment at days 0–6.

#### Ex vivo degradation and stabilization of cfDNA

To trace cell-independent degradation of cfDNA, supernatants from three mESC lines were collected as described above, and incubated for 0, 4, 5, or 24 h at 37 °C without cells. Next, cfDNA was isolated and size-profiled as described. To showcase the degradation of external material, gDNA was added to supernatants prior to ex vivo incubation. To inhibit nucleases, supernatants were either incubated at 70 °C for 30 min or mixed with EDTA (Invitrogen, 15575020) at a final concentration of 25 or 50 µM prior to ex vivo incubation.

#### Microscopy

At day 20, a subset of 3D bioreactor hepatocytes was collected from the bioreactor and transferred to 8-well Ibidi glass bottom plates. Stainings were performed as previously described [22], with Triton X-100 in blocking solution being increased to 0.3%, and nuclei being stained with Hoechst 33342 (Thermo Fisher, #62249, final 10 µg/mL) and primary antibodies for Albumin (Bethyl Laboratories, A80-229A at 1:100) and the secondary antibody (Invitrogen, at 1:1000). Imaging was performed on a CellDiscoverer7-LSM 900 confocal (Zeiss) using ZEN software (Zeiss).

#### Whole-genome bisulfite sequencing (WGBS) of cfDNA

gDNA and matched cfDNA were used as input. gDNA, but not cfDNA, was fragmented using a Covaris S2 system (intensity:10, duty cycle: 5%, cycles per burst: 200, duration: 90 s), cleaned up with the DNA Clean and Concentrator-5 kit (Zymo, D4004), quantified and quality-controlled on a TapeStation device as described above, and 100 ng of sheared reference gDNA and 48 ng of cfDNA were bisulfite converted using the EZ DNA Methylation-Gold kit (Zymo, D5005), eluting in lowEDTA TE buffer, pH 8.0 (IDT11-05–01–13) as described in [29]. WGBS libraries were prepared with the xGen™ Methyl-Seq DNA Library Prep Kit (IDT, 10009824), at 8 indexing PCR cycles with the xGEN UDIPrimer Plate 2, 8nt (IDT, 10009816), and sequenced as 150-bp paired-end reads on an Illumina NovaSeq 6000 system.

#### ONT-SFM sequencing

cfDNA libraries were prepared from cfDNA with the Native Barcoding Kit 24 V14 (Oxford Nanopore, SQK-NBD114.24) according to the manufacturer’s recommendations (https://nanoporetech.com/document/ligation-sequencing-v14-human-cfdna-multiplex-sqk-nbd114-24). Four to five samples per pool were sequenced on a FLO-PRO114M flow cell (Oxford Nanopore). gDNA reference samples were sheared with the Megaruptor3 (Diagenode, B06010003) using the native Barcoding Kit 96 V14 (Oxford Nanopore, SQK-NBD114.96, https://nanoporetech.com/document/ligation-sequencing-gdna-native-barcoding-v14-sqk-nbd114-96), according to the manufacturer’s recommendations, but doubling the initial input DNA amount to a total of 24 µl and adjusting downstream volumes. Four to eight samples per pool were sequenced on a FLO-PRO114M flow cell (Oxford Nanopore).

### Computational analyses

#### WGBS data processing

WGBS data processing was performed as previously described [29, 30]: Briefly, reads were trimmed with cutadapt [31] (v. 4.4; parameters as follows: –quality-cutoff 20 –overlap 5 –minimum-length 25) and Illumina TruSeq adapters were clipped from both reads. Next, 10 and 5 nucleotides were removed from the 5’ and 3’ end (first read), and 15 and 5 nucleotides were removed from the 5’ and 3’ end of (second read). Trimmed reads were aligned to the mm10 genome with BSMAP [32] (v. 2.90; parameters: -v 0.1 -s 16 -q 20 -w 100 -S 1 -u -R). Duplicates were removed using the MarkDuplicates command from GATK [33] (v. 4.4.0.0; with parameters as follows: –VALIDATION_STRINGENCY = LENIENT –REMOVE_DUPLICATES = true). Methylation rates were called with MOABS [34] (v. 1.3.2)’s mcall command. Analyses were restricted to autosomes, only CpGs covered by at minimum 10 and at maximum 150 reads passed criteria for downstream analyses.

#### Oxford Nanopore (ONT) data processing

ONT signal data were base-called with dorado [23] (v. 0.8.3) '5mCG_5hmCG' model and sup mode. Reads were aligned to human chm13v.2 with minimap2 [35] (v. 2.26), with options minimap2 –secondary = no −2 -a -y -t 32 -K 2G -x map-ont. Methylation bed files were generated with the modkit [36] (v. 0.5.1) pileup command using –cpg –combine-strands –threads 40 –filter-threshold 0.8 –ignore h to analyse individual replicates or –cpg –combine-strands –threads 40 –filter-percentile 0.21 –ignore h for aligned bam files of merged replicates. Analyses were restricted to autosomes, only CpGs covered by a minimum of 2 and a maximum of 150 reads passed criteria for downstream analyses. The –ignore h flag was omitted when hydroxymethylation was included in the analysis.

#### Read and fragment length analyses

For Nanopore reads, read length statistics were generated with “dorado summary –verbose”. R”; and the sequence_length_template values for all reads with a minimum mean qscore template value of 10 were plotted in python (v. 3.10.8). For replicate-resolved fragment statistics, histograms were generated with pycoQC (v. 2.5.2), exported to json, and plotted in python (v. 3.10.8).

#### Read coverage karyograms

BAM coverage karyograms were generated with the R package karyoploteR (v. 1.30.0) using the kpPlotDensity command.

#### DNA methylation analysis

*Feature annotation*. For every sample, the arithmetic mean was calculated across features (tiles, enhancers) with bedtools [37] (v. 2.30.0) intersect and groupby. A feature was considered only if at least three CpGs were covered within a region. Whole genome tiles for chm13v.2 and mm10 were generated with bedtools’ makewindows command. *Violin plots, Smooth scatter correlation plots, and principal component analysis*. Violin plots and Smooth scatter plots were generated from raw methylation bed files in R (v. 4.4.1) with vioplot (v. 0.5.0) or the smoothScatter function (euclidean distance). Only regions covered by all samples were utilized for display. PCA analysis and plotting was performed in python (v. 3.10) with scanpy [38] (v. 1.11.0). *Methylation browser tracks*. Integrative Genomics Viewer [39] (v. 2.16.0) was used to generate browser track views from bigwigs previously generated with bedGraphToBigWig (v. 4) from the UCSC Genome Browser's utilities [40]. *Methylation offset plots and delta calculation*. Line plots were generated using extracting the mean CpG methylation value for a feature or globally in with the pandas python package (v. 2.2.3), the difference between cfDNA and gDNA was calculated and the average of the absolute difference (average delta) was determined and displayed in the figure. *Hepatocyte enhancers*. Hepatocyte cell type marker regions and putative hepatocyte enhancers were retrieved from [4] via “wget https://static-content.springer.com/esm/art%3A10.1038%2Fs41586-022-05580-6/MediaObjects/41586_2022_5580_MOESM5_ESM.zip” and “wget https://static-content.springer.com/esm/art%3A10.1038%2Fs41586-022-05580-6/MediaObjects/41586_2022_5580_MOESM6_ESM.zip”, extracted, and hg19 annotations were lifted to chm13v.2 with UCSC liftOver. To trace de-methylation dynamics in maturing cells derived from human iPSCs, markers and enhancers already depleted of DNA methylation in iPSCs (average CpG methylation < = 0.1) were removed from the set. Reversely, to ensure capture of hepatocyte-specific de-methylation, markers and enhancers were required to display low methylation in adult primary human hepatocytes (average CpG methylation < = 0.1 in primary human hepatocytes). Annotations were performed as described above. *Heatmaps and hierarchical clustering*. Clustermaps were generated from raw methylation bed files in python (v. 3.10.8) with seaborn’s (v. 0.13.2) clustermap function (euclidean distance). Only regions covered by all samples were utilized for display.

#### FACS analyses

All FACS analyzes were performed in with BD FACSDiva (version 8.0.1.1) and plotting was performed with python (version 3.10).

#### Image analysis

Downstream image processing of 3D bioreactor hepatocytes was performed Zen Pro and arivis Pro (both Zeiss) to generate 3D reconstructions, subset image creation and scale annotation was next performed in in ImageJ (v. 1.54G).

#### Statistics and reproducibility

N numbers refer to biological replicates or number of experiments performed*,* unless otherwise specified when indicating number of CpGs or specific regions (such as 1 kb tiles or enhancers). Pearson correlation coefficients and p values were calculated with the scipy.stats (v. 1.15.1) python package’s function pearsonr. No statistical measure was performed to determine sample size. Experimenters were not performed blinded.

## Supplementary Information

Additional file 1. File containing Tables S1-S3. Table S1: Doubling time calculations for 3 mESC lines, related to Fig. 1b and Extended Data Fig. 1c-d. Table S2: DNA amounts for 3 mESC lines, related to Fig. 1b and Extended Data Fig. 1c-d. Table S3: DNA amounts for 3 mESC lines treated with DNMT1-inhibitor, related to Fig. 1c-g.

Additional file 2. File containing Figure S1. Figure S1: Gating strategy for FACS analysis of AnnexinV-positive cells.

Additional file 3. File containing Extended Data Figures 1-3. Extended Data Figure 1: Technical controls and characterization of cfDNA from mouse ESC cultures Extended Data Figure 2: Validation and performance metrics of cfDNA-based methylation profiling in 3D bioreactor hepatocyte differentiation. Extended Data Figure 3: Replicate-resolved extended metrics for cfDNA-based methylation profiling in 3D bioreactor hepatocyte differentiation.

## Data Availability

Raw sequencing data are deposited at GEO under the accession GSE293866 [41]. All scripts used, including source data required to reproduce figures, are available under (https://github.com/anjahess/InvitroLiquidBiopsy) [42]. An archived version is provided under (https://doi.org/10.5281/zenodo.18410946) [43]. Microscopy data has been deposited in FigShare and is available under (10.6084/m9.figshare.31173646) [44]. DNA content values for reference in supplementary material file 3 were derived from [45].
