# Supplementary material for: Non-disruptive in vitro monitoring of cellular states with cell-free DNA methylation
Source: Genome Biol. 2026 Feb 17;27:59. doi: 10.1186/s13059-026-03996-1 (PMC12931013; doi:10.1186/s13059-026-03996-1)
Supplement: Supplementary file 2 — Additional file 2. File containing Figure S1. Figure S1: Gating strategy for FACS analysis of AnnexinV-positive cells. [file 13059_2026_3996_MOESM2_ESM.pdf]

Fig. S1 Gating strategy for FACS analysis

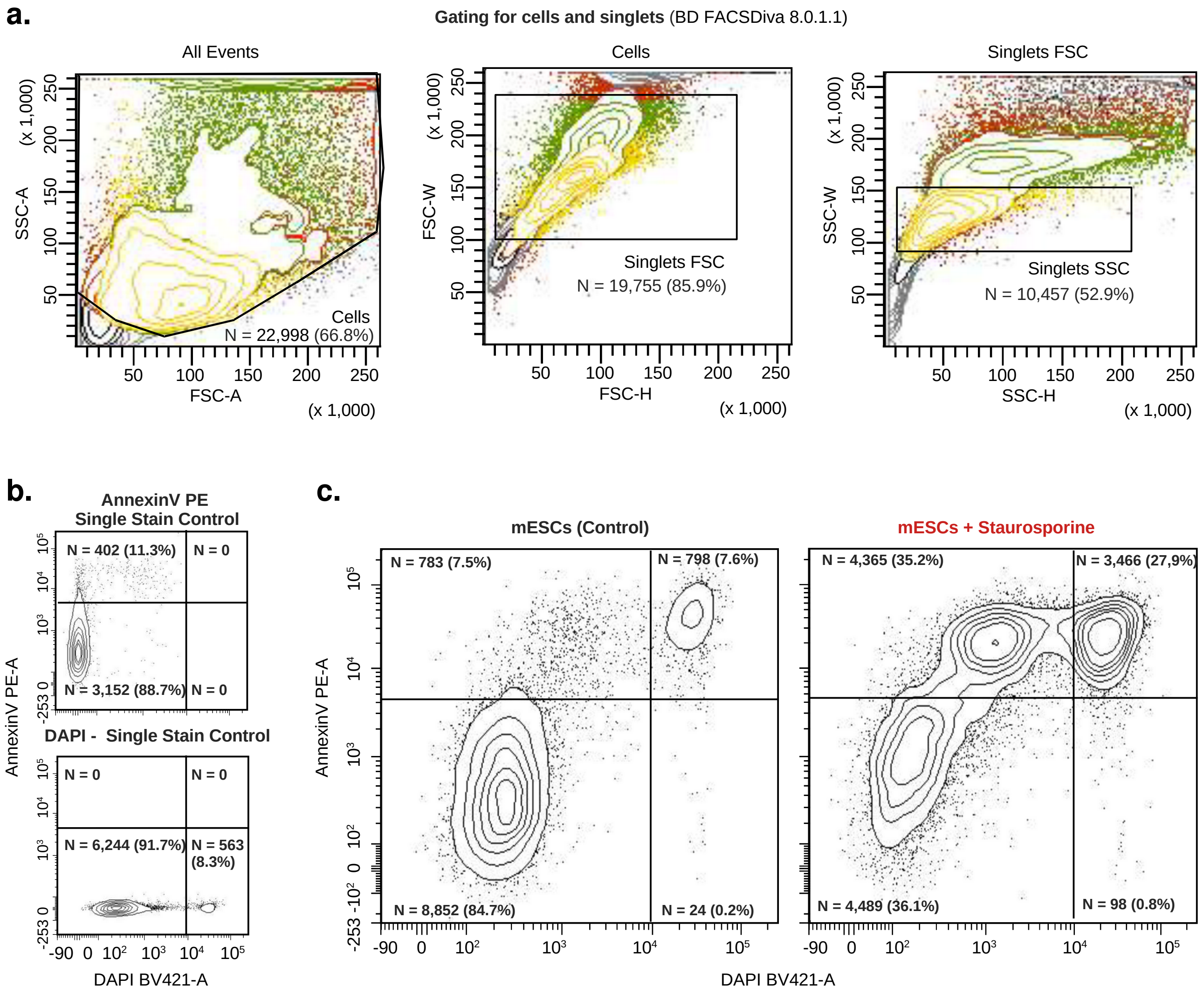

Fig. S1 FACS Gating strategy. a. Scatter plots exemplifying the gating strategy for FACS analysis. Cell-sorting strategy (left), FSC-based doublet exclusion strategy (center), and SSC-based doublet exclusion strategy (right). **b.** Contour plots with outliers to present the strategy to set the fluorophore gates for AnnexinV-PE and DAPI based on signal retrieved from single-stain controls. Top: Annexin V-PE to discriminate apoptotic and non-apoptotic cells; bottom: DAPI to discriminate live and dead cells. **c.** Example contour plots with outliers of cells gated for AnnexinV-PE and DAPI to discriminate live, dead, and apoptotic populations. Quadrant gates, event numbers, and percentage of parent population are annotated in the plot.
