# Supplementary material for: Non-disruptive in vitro monitoring of cellular states with cell-free DNA methylation
Source: Genome Biol. 2026 Feb 17;27:59. doi: 10.1186/s13059-026-03996-1 (PMC12931013; doi:10.1186/s13059-026-03996-1)
Supplement: Supplementary file 3 — Additional file 3. File containing Extended Data Figures 1-3. Extended Data Figure 1: Technical controls and characterization of cfDNA from mouse ESC cultures Extended Data Figure 2: Validation and performance metrics of cfDNA-based methylation profiling in 3D bioreactor hepatocyte differentiation. Extended Data Figure 3: Replicate-resolved extended metrics for cfDNA-based methylation profiling in 3D bioreactor hepatocyte differentiation. [file 13059_2026_3996_MOESM3_ESM.pdf]

Additional file 3: Extended Data Figures

to the publication

"Non-disruptive in vitro monitoring of cellular states with cell-free DNA methylation"

Anja Hess[1,2], Alexander Kovacsovics[1*], Fabian Bachinger[1,3*], Ludovic Vallier[1,3], Helene Kretzmer[1,4], and Alexander Meissner[1]

1. Max Planck Institute for Molecular Genetics, Berlin, Germany
2. Department of Biology, Chemistry and Pharmacy, Freie Universität Berlin, Berlin, Germany
3. Berlin Institute of Health, BIH Center for Regenerative Therapies, Charité-Universitätsmedizin, Berlin, Germany
4. Digital Health Cluster, Hasso Plattner Institute for Digital Engineering, Digital Engineering Faculty, University of Potsdam, Potsdam, Germany

*contributed equally: Alexander Kovacsovics and Fabian Bachinger

Correspondence: A.M. meissner@molgen.mpg.de

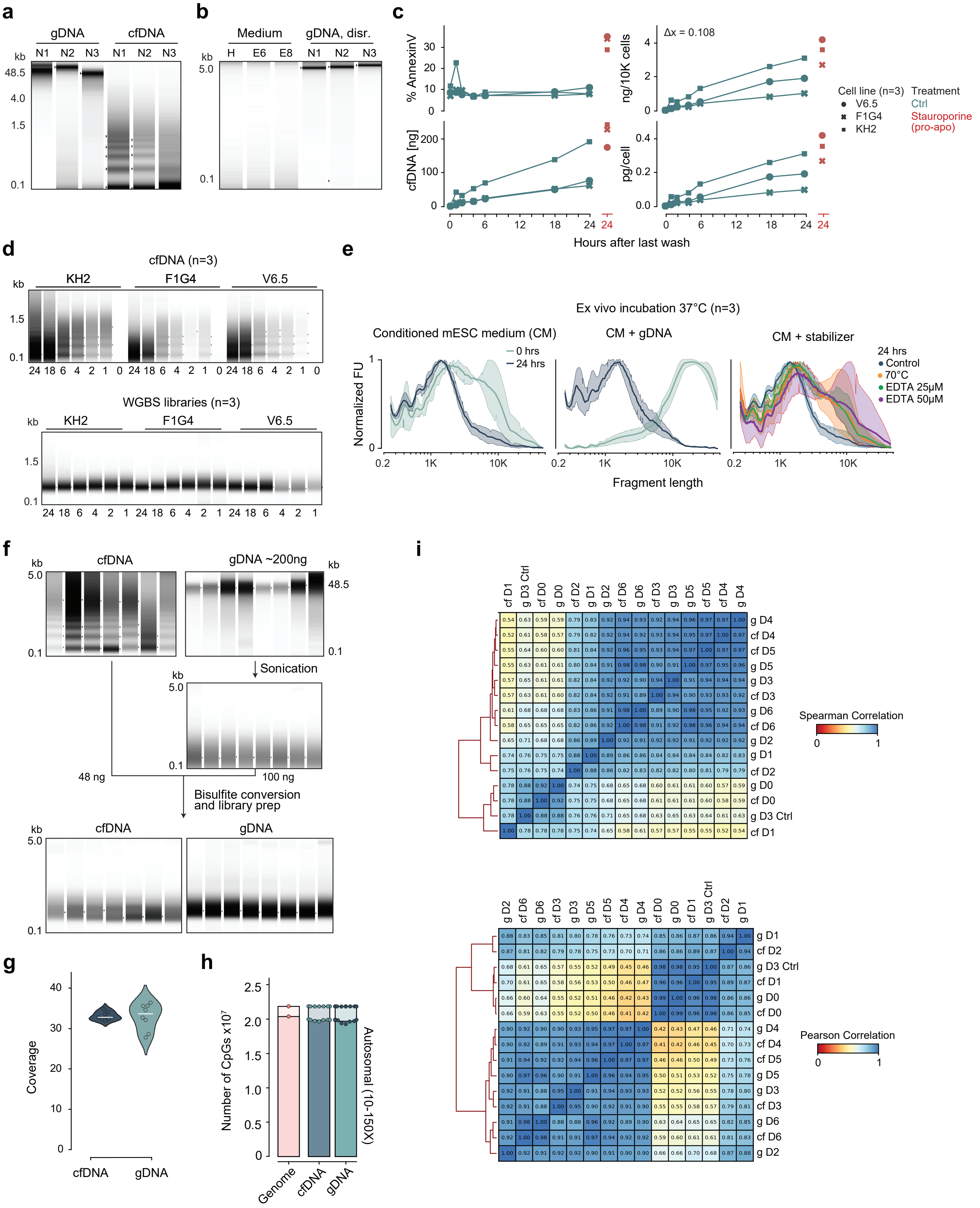

Hess, Extended Data Figure 1

Extended Data Fig. 1: Technical controls and characterization of cfDNA from mouse ESC cultures

a) Representative electrophoresis traces comparing gDNA and cfDNA from mouse ESCs, showing differential fragment sizes of the two DNA types. N = 3.
b) Electrophoresis traces of cfDNA from various medium-only controls (H: Hepatozyme medium; E6: E6 medium; E8: E8 medium (left), and gDNA from mechanically disrupted cells (disr., right). Mechanical disruption of cells produced high-molecular weight DNA (~5 kb), unlike nucleosomally fragmented cfDNA. N = 3.
c) Quantitative metrics from the 24-hour time course (related to Fig. 1b) for each mouse ESC line. Line plots show the FACS-quantified apoptotic fractions (by AnnexinV staining), total cfDNA yield (ng), cfDNA yield normalized by 10,000 cells (ng), and cfDNA yield normalized to total number of cells (pg). Slope (Δx) indicates average cfDNA release per 10,000 cells per hour. Treatment with the pro-apoptotic agent Staurosporine (S) was included as a positive control (red). For reference: the theoretical haploid mouse genome content is 3 pg [45]. N = 3.
d) Electrophoresis traces of cfDNA at 1 to 24 hours post medium change, and corresponding WGBS libraries (related to Fig. 1b). The 0 hour time point, indicating medium conditioned only for a few seconds, serves as the negative control. N = 3.
e) Fragment length distribution of ex vivo incubated mouse ESC cfDNA in various media, showing nuclease-mediated degradation of cfDNA. Conditioned Medium (CM) was incubated at 37°C without cells, and cfDNA fragment sizes were analyzed at 0 and 24 hours (left). Additional conditions included the addition of gDNA to the medium (middle), or nuclease inactivation by heat (70 °C) or EDTA (25 µM or 50 µM, right). Marker peak at 100 bp cropped from the data for visualization. Only nuclease-inhibited samples retained higher molecular weight fragments (~4–12 kb), whereas the control showed degradation towards low molecular weight fragments (1–4 kb). N = 3.
f) Library preparation workflow for cf- and gDNA WGBS (related to Fig. 1c-g), showing gel electrophoresis profiles. Note: gDNA was quantified before and after sonication, and 100 ng input refers to the amount measured after sonication. cfDNA required no sonication.
g) Violin plots show genome-wide coverage for cfDNA and gDNA WGBS libraries (related to Fig. 1c-g).
h) Barplots with overlaid samples (dots) showing the number of CpGs covered in cf (blue) and matched gDNA (green) WGBS libraries (related to Fig. 1c-g), compared to the genome wide number of CpGs (mm10, light pink). Lighter areas indicate filtered CpGs (sex-chromosome or a coverage below 10 or above 150X).
i) Spearman (top) and Pearson (bottom) correlation plots and related hierarchical clustering by correlation coefficients based on global DNA methylation for all samples related to Fig. 1c-g. Color represents the numerical value of each correlation coefficient. Each individual correlation coefficient is also denoted in black inside the diagram.

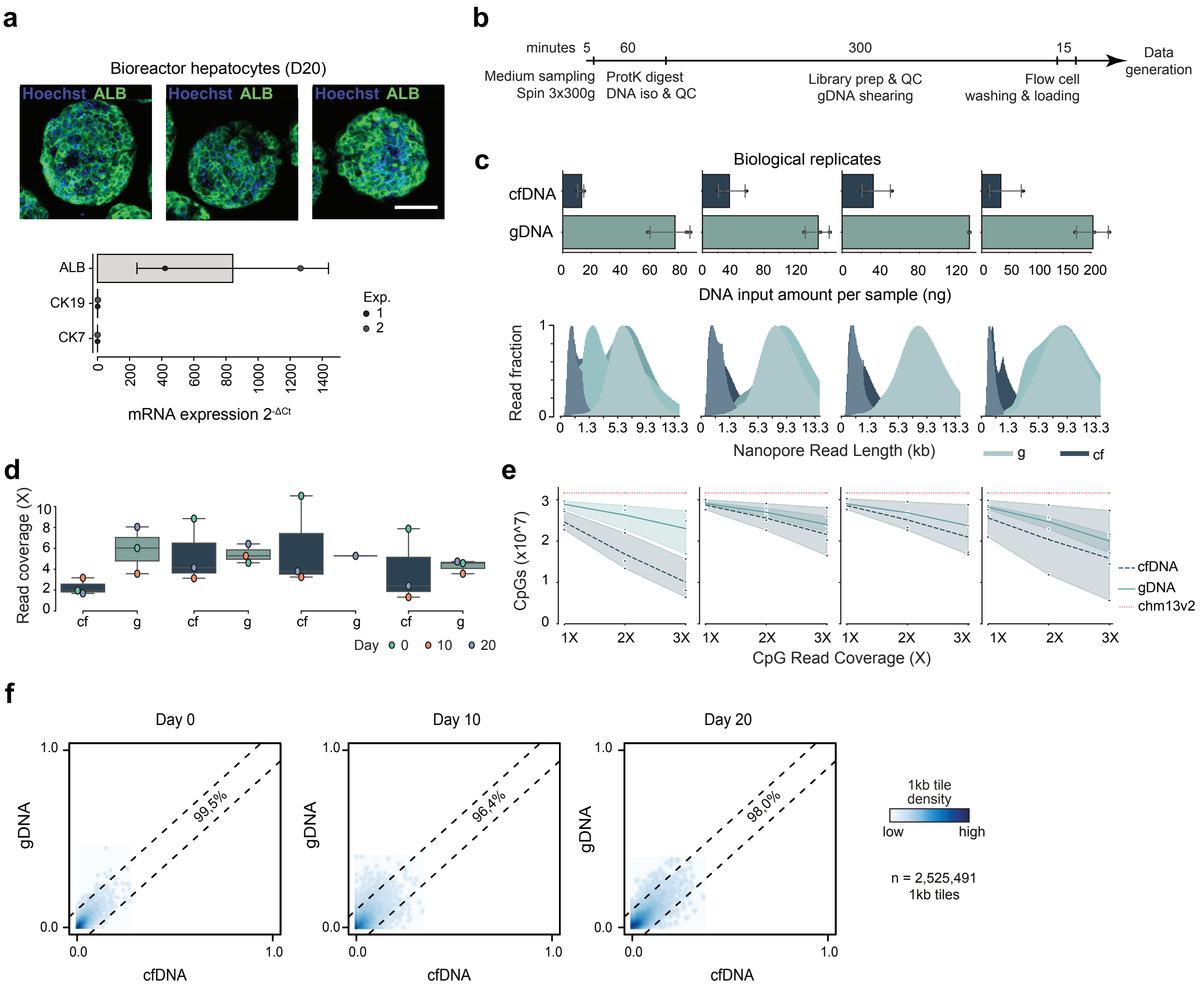

Hess, Extended Data Figure 2

Extended Data Fig. 2: Validation and performance metrics of cfDNA-based methylation profiling in 3D bioreactor hepatocyte differentiation.

a) Functional validation of 3D bioreactor hepatocytes. Immunofluorescence (top) and qPCR data (bottom) confirm Albumin expression at day 20. IF: Hoechst, blue; nuclei and Albumin, green; scale bar: 100 µm. Representative 3D hepatocyte structures are shown. N = 3 (IF), n = 2 (qPCR).
b) Schematic of a one-day workflow from cfDNA collection to Nanopore sequencing data generation. Time per step is indicated on top.
c) Top: Barplots show average DNA input amounts for cfDNA and gDNA Nanopore libraries for each biological replicate. Each column corresponds to an individual replicate. Dots denote individual samples, error bars denote the 95% percentile interval. N = 4 cfDNA, n = 3-4 gDNA. Bottom: Read length histograms show the fragment length by the relative fraction of reads for each biological replicate from the multiplexed Nanopore libraries. Individual sample traces are plotted as overlays, represented by varying color shades. N = 4 cfDNA, n = 3-4 gDNA.
d) Box plots with overlaid dot plots show the average genomic read coverage of aligned reads for each biological replicate. Boxes show data quartiles, and whiskers extend to the rest of the distribution. Individual samples are shown as dots, dot colors denote the day of the hepatocyte differentiation (green – Day 0; orange – Day 10, blue – Day 20). N = 4 cfDNA, n = 3-4 gDNA.
e) Line plots with overlaid scatter plots show the autosomal CpG coverage for each biological replicate at varying read-depth thresholds, with each dot representing an individual sample. Striped red line on the top indicates the number of autosomal CpGs in the human chm13v.2 reference genome. Band displays the entire distribution of the data. The color denotes the DNA type (blue – cfDNA, green – gDNA). N = 4 cfDNA, n = 3-4 gDNA.
f) Genome-wide correlation plots for showing averaged hydroxymethylation levels per 1kb tile. Data were reanalyzed as follows: Methylation bed files were generated from chm13v2-aligned bam files of merged replicates with modkit's pileup command using --cpg --combine-strands --threads 40 --filter-percentile 0.21, and next, hydroxy- and methyl calls were separated, and samples were analyzed at a 2X read coverage threshold. Plots were generated from 10,000 randomly selected tile regions for representation, concordance values of ± 10% intervals were calculated on all regions and are denoted inside the striped lines. N = 3.

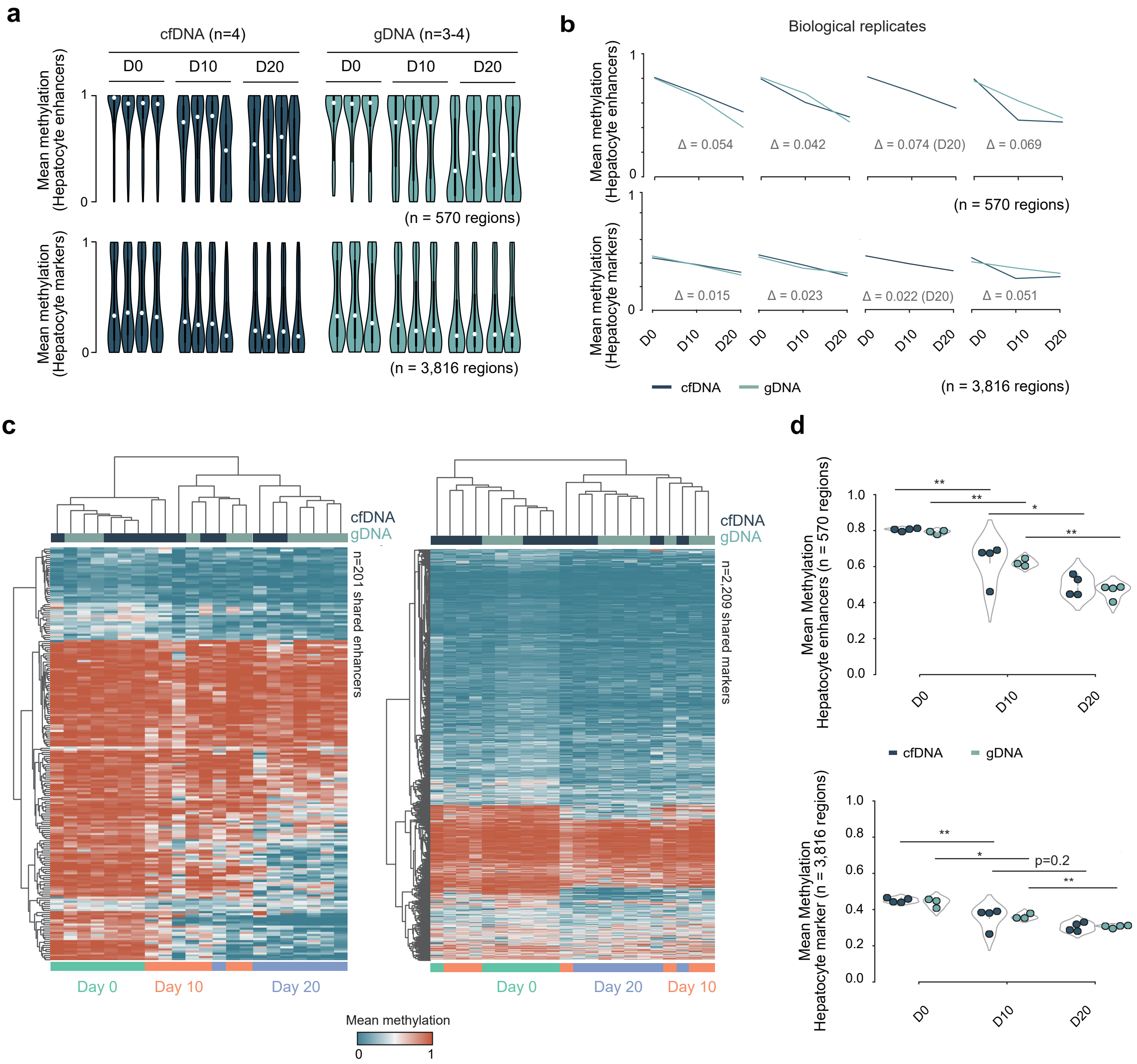

Hess, Extended Data Figure 3

Extended Data Fig. 3: Replicate-resolved extended metrics for cfDNA-based methylation profiling in 3D bioreactor hepatocyte differentiation.

a) Violin plots of mean CpG methylation in 570 hepatocyte enhancers (top) and 3,816 hepatocyte cell type marker regions (bottom) across the time course for cfDNA and gDNA (adjusted to iPSCs, based on [4]). Each violin represents an individual sample across the biological replicates. N = 4 cfDNA, n = 3-4 gDNA.

b) Line plots show mean CpG methylation over time in cfDNA and gDNA. Mean differences between conditions are indicated. Data related to a, each column represents an individual replicate. N = 4 cfDNA, n = 3-4 gDNA.

c) Heatmaps with hierarchical clustering of mean CpG methylation in shared hepatocyte enhancers (n = 201) and cell type marker regions (n = 2,209) across the time course for cfDNA and gDNA per each individual sample across all biological replicates. Only regions covered in all samples are displayed. N = 4 cfDNA, n = 3-4 gDNA.

d) Violin plots with overlaid scatter plots display the average methylation levels per biological replicate for 570 hepatocyte enhancers (top) and 3,816 hepatocyte markers (bottom). One-Way ANOVA followed by a Conover's posthoc test across groups has been performed for statistical comparison and results are highlighted: ** $p<0.01$; * $p<0.05$. N = 4 (cfDNA), N = 3-4 (gDNA).
